# Quantifying Female Waist Aesthetics: Preferences for Waist-to-Hip Ratio and Abdominal Definition

**DOI:** 10.1093/asjof/ojag138.010

**Published:** 2026-07-24

**Authors:** D Colton Riley, Javier S Hernaez, Peter Granger

**Affiliations:** Mayo Clinic, Jacksonville Florida; University of Navarra Schoole of Medicine, Pamplona Spain; Mayo Clinic, Jacksonville Florida

## Abstract

**Purpose:**

The female waist and torso remain fundamental aesthetic elements in body contouring, yet empirical data describing public preferences for waist-to-hip ratio (WHR) and abdominal muscle definition are limited. Misalignment between patient perceptions and actual aesthetic preferences may influence body dissatisfaction, drive unnecessary procedures, and shape unrealistic surgical goals. This study aimed to (1) evaluate how females perceive their own waist shape and ideal appearance using standardized manipulated images, (2) quantify male preferences for female WHR and abdominal definition, and (3) assess the discrepancy between what women believe men find attractive and men’s actual preferences.

**Methods:**

An anonymous online survey was distributed internationally and collected 900 responses from adult participants. Two digitally manipulated, standardized female torso images were created: one varying waist-to-hip ratio across five levels (WHR 0.60 to 0.80) and one varying abdominal muscle definition. Male participants rated which WHR and abdominal definition they found most attractive. Female participants reported (1) which WHR and definition most resembled their current appearance, (2) which represented their ideal, and (3) which they believed men found most attractive. Additional demographic and body image variables were collected. Descriptive statistics were generated, and comparative analyses (chi-square tests) were conducted to evaluate differences in preference distributions and female perception versus male reality.

**Results:**

A total of 900 respondents participated, including 41% men and 59% women. Male respondents showed a clear preference for smaller waist-to-hip ratios (WHR). The lowest WHR (0.60) was the most frequently selected option (38%), with steadily decreasing selections as WHR increased. Men also most preferred mild abdominal definition (Option B). Female responses revealed notable perception gaps: women most often perceived themselves as having midrange WHRs (0.70) but selected lower WHRs (0.65) as ideal, and they substantially overestimated men’s preference for the smallest WHR. Subgroup analyses showed that male WHR preferences varied significantly by continent (p < 0.05), with Europe, North and South America favoring smaller WHRs, while Africa, Australia, and Asia preferred larger ones. Higher male physical activity was associated with selecting smaller WHRs (r = –0.220, p < 0.05) and slightly greater abdominal definition (r = 0.169, p < 0.05). Social media use did not influence male preferences. Among women, physical activity strongly predicted desired abdominal muscularity (r = 0.236, p < 0.05) but not ideal WHR. Perceived social media influence significantly shaped ideal WHR (r = 0.202, p < 0.05), such that women who reported more negative influence selected smaller, more exaggerated WHRs. Time spent on social media did not significantly affect female aesthetic preferences.

**Conclusion:**

This study provides quantitative evidence describing public preferences for female waist and torso aesthetics, revealing important divergences between perception and reality. Men strongly prefer smaller waist-to-hip ratios and mild abdominal definition, but not to the extreme levels that women commonly assume. Women’s own ideal waist shape aligns directionally with male preferences, yet women significantly overestimate the degree to which men favor the smallest possible WHR. This misperception may contribute to body dissatisfaction, drive interest in unnecessary procedures, or set unrealistic surgical expectations.

For aesthetic surgeons, these findings highlight the need for patient-centered counseling that addresses perception gaps, corrects inaccurate beliefs about male preferences, and reinforces natural, proportionate outcomes rather than extreme contouring. The data support a trend toward moderate enhancement rather than highly exaggerated WHR reduction or aggressive abdominal definition in female body contouring. Incorporating these insights may improve patient satisfaction, promote psychological well-being, and guide surgical planning grounded in evidence-based aesthetics.

